# Chemoselektive γ‐Oxidation von β,γ‐ungesättigten Amiden mit TEMPO

**DOI:** 10.1002/ange.202104023

**Published:** 2021-07-20

**Authors:** Sebastian Heindl, Margaux Riomet, Ján Matyasovsky, Miran Lemmerer, Nicolas Malzer, Nuno Maulide

**Affiliations:** ^1^ Institut für Organische Chemie Universität Wien Währinger Straße 38 1090 Wien Österreich

**Keywords:** Amide, Chemoselektivität, Oxidation, Radikalreaktion, Regioselektivität

## Abstract

Ein chemoselektives und robustes Protokoll zur γ‐Oxidation von β,γ‐ungesättigten Amiden wird dargelegt. Bei dieser Methode ermöglicht elektrophile Amidaktivierung eine bei ungesättigten Amiden bisher selten angewendete regioselektive Reaktion mit TEMPO, die zu γ‐aminoxylierten α,β‐ungesättigten Amiden führt. Radikalische Zyklisierungen und Oxidationen der synthetisierten Produkte untermauern die Nützlichkeit der hergestellten Verbindungen.

## Graphical abstract

Eine direkte γ‐Oxidation β,γ‐ungesättigter Amide wird beschrieben. Diese regioselektive Reaktion mit TEMPO verläuft unter milden Bedingungen und toleriert unter anderem verschiedene Carbonylfunktionalitäten. Präsentiert wird ein seltenes Beispiel von regioselektiver Amidaktivierung an ungesättigten Substraten, das in oxyaminierten Produkten resultiert. Die Produkte eignen sich unter anderem für bemerkenswert selektive radikalische Zyklisierungen.

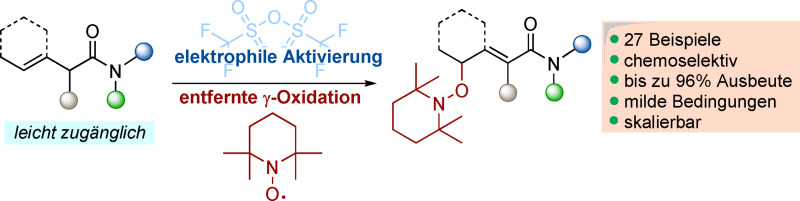

Die träge Reaktivität von Amiden mit klassischen Nukleophilen, die dem elektronenspendenden Stickstoff zuzuschreiben ist, ist bereits gut dokumentiert. Um Reaktivität am Carbonyl zu generieren, ist es daher oft nötig, diese Substanzfamilie zu aktivieren.^[1]^ Nach frühen, erfolgreichen Versuchen der Amidaktivierung^[2]^ hat sich schließlich Trifluormethansulfonsäureanhydrid (Tf_2_O) als generelles Aktivierungsreagenz durchgesetzt, nachdem es von Ghosez et al. 1981 erstmals dafür verwendet wurde.[^3^, ^4^, ^5^] Das hat den Weg für viele weitere Entdeckungen geebnet, wie zum Beispiel Movassaghis Heterozyklensynthese, Huangs sequentielle reduktive Alkylierung und Charettes chemoselektive Reduktionsmethoden.[^6^, ^7^, ^8^, ^9^] Unsere Arbeitsgruppe etablierte diese Aktivierungsmethode, um Zugang zu hochreaktiven Keteniminiumionen für die Entwicklung von Umlagerungsreaktionen, wie α‐Arylierungen[^10^, ^11^] und α‐Aminierungen,^[12]^ zu bekommen. In Kombination mit *N*‐Oxid‐Reagentien^[13]^ wurde ein konzeptionell unterschiedlicher Ansatz einer Umpolungsreaktion entwickelt, welcher nukleophile α‐Eingliederung von Halogeniden^[14]^ und anderen Heteroatomen in Amide^[15]^ sowie auch die Bildung von Lactamen^[16]^ und 1,4‐Dicarbonylen ermöglicht.^[17]^

Der Zugang zu entfernteren Positionen im Gebiet der Amidaktivierung blieb, im Vergleich zu dieser Fülle an Methoden zur α‐Funktionalisierung,[^10^, ^11^, ^12^, ^13^, ^14^, ^15^, ^16^, ^17^, ^18^] großteils unerforscht. Zwei Beispiele von γ‐Aminoxylierungen konjugierter Acyloxazolidinon(imid)‐Ti‐Enolaten wurden von Romea und Urpi beschrieben (Schema 1 a).^[19]^ Ein Se‐katalysierter Ansatz für die Synthese von γ‐alkoxy‐ oder γ‐hydroxy‐α,β‐ungesättigten Carbonylverbindungen wurde von Tiecco entwickelt, jedoch wurde nur ein Beispiel eines Amids beschrieben. Ein Überschuss von Ammoniumpersulfat als Oxidationsmittel war nötig, wodurch mögliche funktionelle Gruppen limitiert wurden (Schema 1 b).^[20]^

**Scheme 1:**
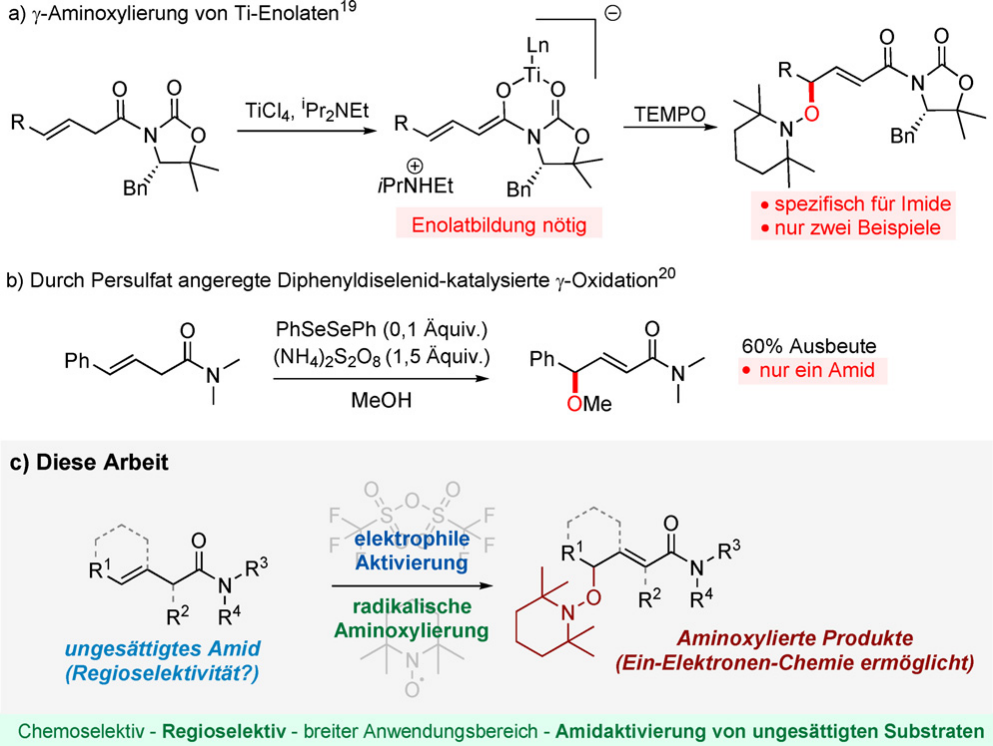
a,b) Strategien für γ‐Oxidation von β,γ‐ungesättigten Amiden. c) Vorgeschlagener Ansatz für die herausfordernde elektrophile Aktivierung von ungesättigten Amiden.

TEMPO‐Addition zu Ketenen wurde bereits früher mit einem Beispiel von γ‐Aminoxylierung demonstriert, jedoch mit niedriger Ausbeute.^[21a]^ γ‐Hydroxylierung von Carbonylverbindungen, jedoch nicht selektiv für Amide, wurde durch Kupferkatalyse ermöglicht.^[21b]^ Eine generelle Methode für direkte, chemoselektive γ‐Oxidation von ungesättigten Amiden ist bisher noch nicht erforscht. Unsere Arbeitsgruppe hat bereits das Abfangen eines Keteniminiumions im oxidativen Kontext über *N*‐Oxide hinaus untersucht, indem das stabile Radikal TEMPO verwendet wurde.[^22^, ^23^] Wir präsentieren hier einen Ansatz zur chemoselektiven γ‐Oxidation von ungesättigten Amiden. Unserem Wissen nach ist das ein unkonventionelles Beispiel für elektrophile Amidaktivierung einerseits aufgrund der Verwendung ungesättigter Substrate, andererseits aufgrund der faszinierenden Reaktivität, die freigesetzt wird, wenn die Reaktionsvielfalt der Ein‐Elektronen‐Prozesse miteingebunden wird.

Zu Beginn fokussierten wir uns auf das β,γ‐ungesättigte Modellamid **1 a**. Solche Substrate kommen selten im Kontext der elektrophilen Amidaktivierung vor, doch überzeugte das Reaktivitätspanorama der selektiven γ‐Oxidation mit begleitender Migration der Doppelbindung hier von Beginn an. Weitere Optimierungen (siehe Zusatzinformation für Details) zeigten, dass knapp über zwei Äquivalente TEMPO für einen effizienten Umsatz nötig sind. Genauso wichtig ist die Wahl der Aufarbeitung. Gesättigte, wässrige NaHCO_3_‐Lösung war optimal und ermöglichte die Isolierung von Produkt **2 a** mit 96 % Ausbeute. Es ist noch hervorzuheben, dass Substrat **1 a** ein leicht zugängliches Produkt einer einfachen dekonjugativen Knoevenagel‐Kondensation mit anschließender Amidkupplung ist (Schema 2, siehe Zusatzinformation für Details).

**Scheme 2:**
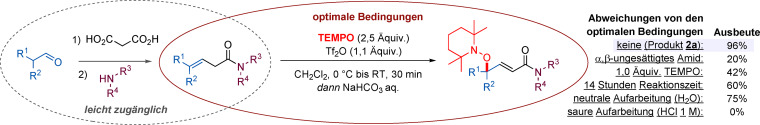
Optimierte γ‐Aminoxylierung von ungesättigten Amiden.

Mit den optimierten Bedingungen erforschten wir den Anwendungsbereich der Reaktion, indem wir zunächst mögliche Substitutionen an der Kohlenstoffkette untersuchten (genauer: verbunden mit dem Olefin, Schema 3). Verschiedene Alkylsubstituenten wurden an der terminalen Position toleriert und lieferten die gewünschten Produkte (**2 a**, **2 b**, **2 c**) in guten bis exzellenten Ausbeuten. Ein endständiges Olefin führte in 63 % Ausbeute zu dem entsprechenden Produkt **2 d**. Sehr erfreut waren wir ob der Entdeckung, dass ein β‐Allen ebenso ein geeignetes Substrat für diese Transformation ist und dadurch das α,β‐γ,δ‐ungesättigte Amid **2 e** in 55 % Ausbeute hergestellt werden konnte. Amide mit einem zweiten β‐Substituenten konnten auch verwendet werden (**2 h**), während jedoch eine α‐Methylgruppe zu verminderter Reaktivität führte (**2 f**).

**Scheme 3:**
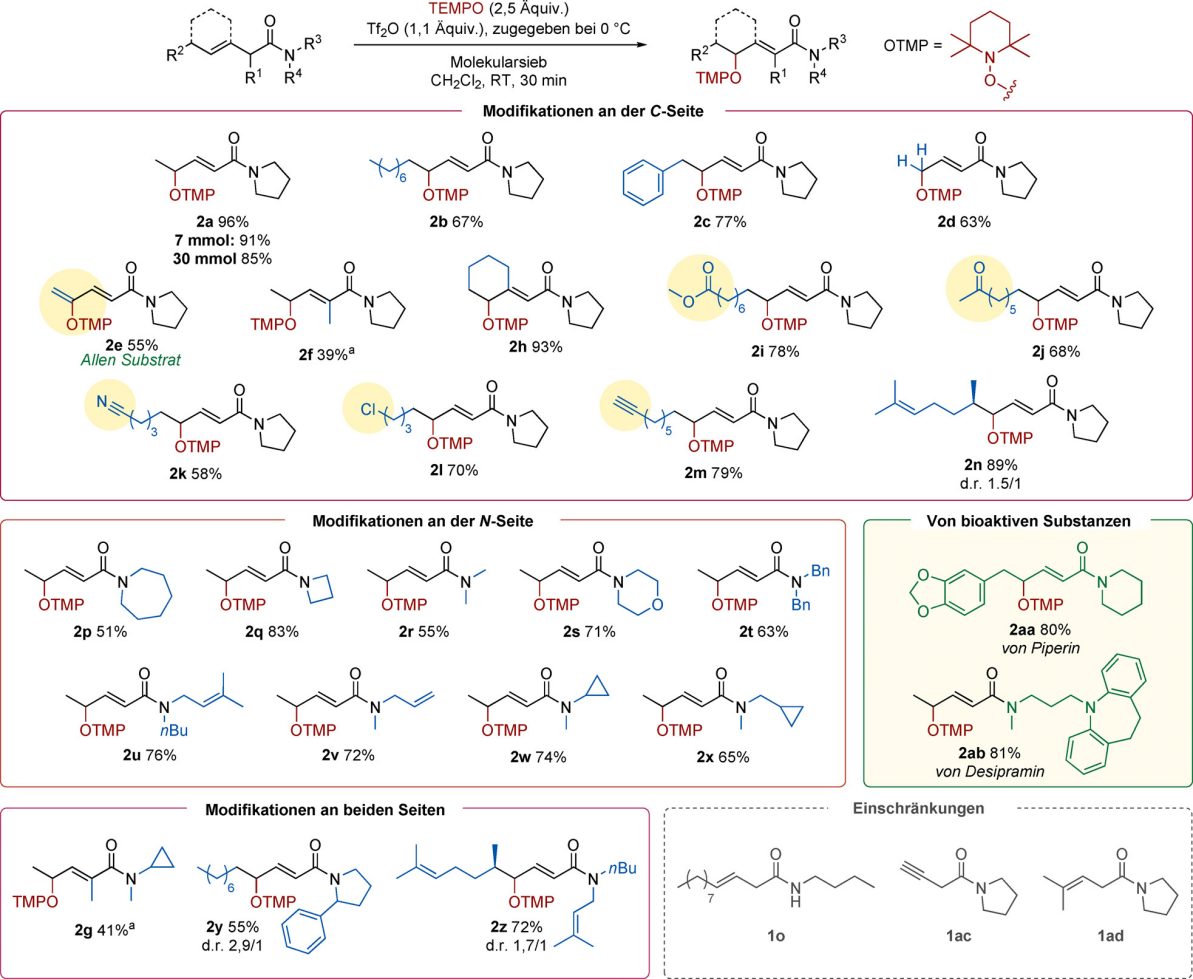
Anwendungsbereich der γ‐Oxidation von ungesättigten Amiden. [a] Reaktionsbedingungen: 40 °C für 4 Stunden.

Generell zeigte diese Reaktion eine sehr gute Toleranz gegenüber funktionellen Gruppen. Ein allgemeines Merkmal der elektrophilen Amidaktivierung ist, dass andere Carbonylfunktionalitäten wie Ester (**2 i**), Ketone (**2 j**) oder Nitrile (**2 k**) unbeeinflusst bleiben, wie auch Halogenide (**2 l**). Zusätzliche ungesättigte Motive (**2 m** und **2 n**) blieben unter den Reaktionsbedingungen ebenso unberührt. Weiters untersuchten wir verschiedene Substituenten am Amid‐Stickstoff. Symmetrische (**2 p**, **2 q**, **2 r**, **2 s**, **2 t**) sowie auch unsymmetrische Amide (**2 u**, **2 v**, **2 w**, **2 x**) wurden toleriert. Im letzten Fall wurden auch keine Zyklisierungsprodukte gefunden, welche von einer radikalischen Addition an ein Olefin oder der Öffnung eines Cyclopropans stammen könnten. Komplexere Substrate wurden auch mit Leichtigkeit γ‐oxidiert (**2 g**, **2 y**, **2 z**). Die Reaktion war auch skalierbar: Mit einem Gramm **1 a** wurde das Produkt ohne Ausbeuteverlust gewonnen, was die Robustheit dieser Methode zusätzlich unterstreicht. Ähnliche Resultate wurden bei einer 100‐fachen Steigerung des üblicherweise verwendeten 0,3‐mmol‐Ansatzes erhalten. Ein sekundäres Amid (**1 o**) oder ein Alkin (**1 ac**) waren keine geeigneten Substrate für diese Bedingungen, und beide Reaktionen führten nur zu Zersetzung. Die Verwendung einer γ,γ‐disubstituierten Verbindung (**1 ad**) verlief ohne Reaktion.

An diesem Punkt fokussierten wir uns darauf, die Reaktivität der neu hergestellten Aminoxylamide zu erforschen (Schema 4). Als **2 a** *m*CPBA ausgesetzt wurde, entstand das Keton **3 a** in 78 % Ausbeute, exklusiv als (*E*)‐Isomer. Die Bildung des (*Z*)‐Isomers (wenn auch in bescheidener Ausbeute) wurde durch Bestrahlen von **2 a** unter O_2_ ermöglicht (Schema 4 a). Die Bildung des 1,4‐Dicarbonyls **3 c** und dessen Derivat **3 d** unterstreicht, dass diese Methode zur schnellen Herstellung bioaktiver Substanzen – in diesem Fall mit antimikrobischen Eigenschaften – eingesetzt werden kann.^[24]^

**Scheme 4:**
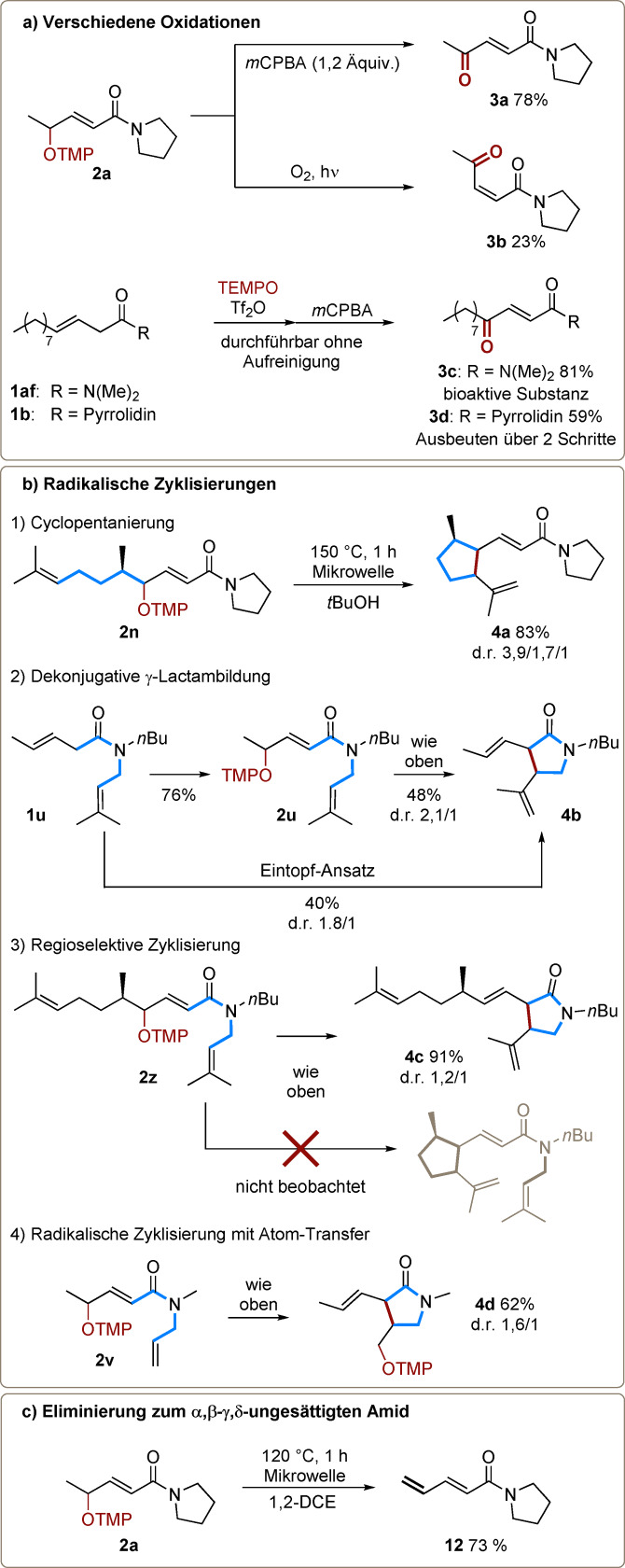
a) Oxidation und Synthese von bioaktiven Substanzen aus einem γ‐OTMP‐α,β‐ungesättigten Amid. b) Thermische 5‐*exo*‐*trig*‐radikalische Zyklisierung von γ‐OTMP‐α,β‐ungesättigten Amiden. c) Eliminierung eines γ‐OTMP‐α,β‐ungesättigten Amids.

Als nächsten Schritt stellten wir die Hypothese auf, dass die Verbindungen **2** für eine homolytische C‐O‐Spaltung zugänglich sind und so ein delokalisiertes allylisches Radikal als Intermediat entsteht.^[25]^ In Anbetracht dessen wurde ein Isoprenyl‐substituiertes Amid als Substrat zur Erforschung des thermisch generierten Radikals herangezogen, da dieses das Radikal durch Zyklisierung abfangen kann (Schema 4). Erfreulicherweise erfolgte die Thermolyse von **2 n** bei 150 °C unter Mikrowellenstrahlung und lieferte das erwartete Produkt **4 a** in 83 % Ausbeute (Schema 4 b‐1). Interessant war weiters die Entdeckung, dass ein Isoprenylsubstituent am Stickstoff, wie bei Amid **2 u**, eine Zyklisierung zur α‐Position eingeht und das γ‐Lactam **4 b** liefert (Schema 4 b‐2). Diese Verbindung konnte auch in einem Eintopf‐Prozess über zwei Schritte ausgehend von Amid **1 u** unter Erhöhung der gesamten Ausbeute hergestellt werden. Bemerkenswert ist, dass diese Produkte augenscheinlich durch 5‐*exo*‐*trig*‐Zyklisierungen mit anschließender oxidativer Eliminierung entstehen, anstatt durch Atomtransfer. Ein kompetitives Experiment mit Amid **2 z** deckte auf, dass die Bildung eines γ‐Lactams die mögliche Zyklisierung der Seitenkette verdrängt und ausschließlich zur Bildung des Produkts **4 c** führt (Schema 4 b‐3). Es ist vorstellbar, dass sowohl Nähe zum elektronenziehenden Carbonyl (das Radikal wird elektrophiler) als auch die Starrheit der Amidbindung (steigert günstige Konformationen) hilfreiche Faktoren für die γ‐Lactambildung sind. Wenn ein monosubstituiertes Olefin (**2 v**) als Akzeptor verwendet wird, entsteht ein Zyklisierungsprodukt durch OTMP‐Transfer (**4 d**) in 62 % Ausbeute (Schema 4 b‐4).[^25a^, ^26^] Wenn kein passender Radikalakzeptor vorhanden ist, wie bei Substrat **2 a** (Schema 4 c), führt die Thermolyse in der Mikrowelle bei 120 °C zur Eliminierung und somit zu Bildung eines α,β‐γ,δ‐ungesättigten Amids (**12**).

Mechanistisch nehmen wir an, dass ein TEMPO‐Radikal das Keteniminiumintermediat **I**[^22^, ^23^] angreift und die radikalische Spezies **II** generiert. Diese rekombiniert rapide mit einem zweiten Äquivalent TEMPO an der distalen γ‐Position, wodurch Intermediat **III** entsteht. Eine solche Reaktion müsste auf einer Radikal‐Radikal‐Kreuzkupplung beruhen, welche durch den anhaltenden Radikaleffekt kontrolliert und ermöglicht wird.^[27]^ Fragmentierung resultiert in Produkt **IV** und dem Imminiumion **V** mit verringerter Ringgröße.^[23]^ Es ist erwähnenswert, dass das entsprechende Amin **VI** nach Behandlung des Rohmaterials mit NaBH_4_ isoliert werden konnte (Schema 5).

**Scheme 5:**
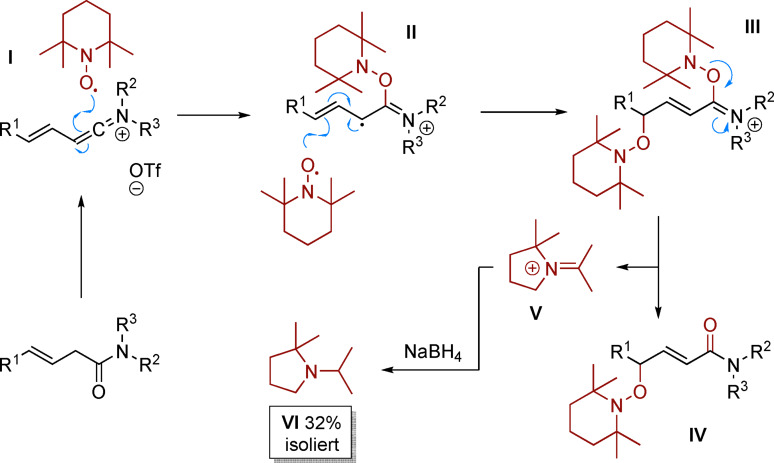
Vorgeschlagener Mechanismus.

Zusammenfassend haben wir eine chemoselektive Methode zur γ‐Oxidation von ungesättigten Amiden mittels elektrophiler Aktivierung unter milden Bedingungen entwickelt. Die gewonnenen Verbindungen ermöglichen eine große Bandbreite an weiterführenden Funktionalisierungen, welche neben Oxidationen die etablierte Aminoxylfunktionalität als Hebel für faszinierende Ein‐Elektronen‐Ringbildungen nutzen. Die Überschneidung von elektrophiler Amidaktivierung mit den einzigartigen Eigenschaften der Ein‐Elektronen‐Chemie garantiert aufregende Wege für die weitere Forschung.

## Conflict of interest

Die Autoren erklären, dass keine Interessenkonflikte vorliegen.

## Supporting information

As a service to our authors and readers, this journal provides supporting information supplied by the authors. Such materials are peer reviewed and may be re‐organized for online delivery, but are not copy‐edited or typeset. Technical support issues arising from supporting information (other than missing files) should be addressed to the authors.

Supporting Information
